# Measuring the Energy of Ventilation and Circulation during Human Walking using Induced Hypoxia

**DOI:** 10.1038/s41598-017-05068-8

**Published:** 2017-07-10

**Authors:** Masahiro Horiuchi, Yoshiyuki Fukuoka, Yoko Handa, Daijiro Abe, Herman Pontzer

**Affiliations:** 1 Division of Human Environmental Science, Mt. Fuji Research Institute, Kami-yoshida 5597-1, Fuji-yoshida-city, Yamanashi, 4030005 Japan; 2 Faculty of Health and Sports Science, Doshisha University, Tatara 1-3, Kyotanabe, Kyoto 6100394 Japan; 3 Center for Health and Sports Science, Kyushu Sangyo University, Matsukadai 2-3-1, Higashi-ku, Fukuoka-city, Fukuoka, 8130003 Japan; 4 Department of Anthropology, Hunter College, The City University of New York, New York, NY 10065 USA; 5 New York Consortium for Evolutionary Primatology, New York, NY 10065 USA

## Abstract

Energy expenditure (EE) during walking includes energy costs to move and support the body and for respiration and circulation. We measured EE during walking under three different oxygen concentrations. Eleven healthy, young, male lowlanders walked on a treadmill at seven gait speeds (0.67–1.83 m s^−1^) on a level gradient under normobaric normoxia (room air, 21% O_2_), moderate hypoxia (15% O_2_), and severe hypoxia (11% O_2_). By comparing the hypoxia-induced elevation in heart rate (HR [bpm]), ventilation (V_E_ [L min^−1^]) with the change in energy expenditure (EE [W]) at each speed, we were able to determine circulatory and respiratory costs. In a multivariate model combining HR and V_E_, respiratory costs were 0.44 ± 0.15 W per each L min^−1^ increase in V_E_, and circulatory costs were 0.24 ± 0.05 W per each bpm increase in HR (model adjusted r^2^ = 0.97, p < 0.001). These V_E_ costs were substantially lower than previous studies that ignored the contribution of HR to cardiopulmonary work. Estimated HR costs were consistent with, although somewhat higher than, measures derived from catheterization studies. Cardiopulmonary costs accounted for 23% of resting EE, but less than 5% of net walking costs (i.e., with resting EE subtracted).

## Introduction

Humans’ bipedal walking gait is a defining feature of our lineage, and the metabolic costs of walking have figured prominently in discussions of human evolution and ecology^1–3^. The energy cost of walking must include energy expenditure (EE) to move and support the body and to fuel physiological changes, such as increased ventilation and circulation, needed to meet increased metabolic demand. To date, studies of walking cost have largely ignored the costs of ventilation and circulation, focusing instead on the factors known to affect the costs of movement such as walking speed, internal and external work^4, 5^, gradients^6, 7^, body mass^8–11^, and load carriage^12–15^. Locomotor cost is of central importance in human ecology^16, 17^, behavior^18, 19^, evolution^1–3^, and biomechanics research^4, 5, 7, 12^, and a full accounting of human walking cost and its determinants requires an understanding of the contribution of cardiopulmonary work to total metabolic cost.

Respiratory costs have been measured in humans and other vertebrates during rest and exercise^20–30^. Early studies of respiratory cost at rest, measured by having the subject breathe through a long tube to increase the dead space and thus the work needed to breathe, found that ventilation costs in humans at rest were quite low (<1 mlO_2_ per L V_E_)^21, 27^. Subsequent studies reported substantially greater respiratory costs during exercise, though still less than 10% of total exercise energy expenditure^20, 22, 23, 25, 29, 30^. However, these studies have generally used an experimental design in which respiratory costs are not measured during exercise, but rather during voluntary hyperpnea in which subjects match their respiratory frequency and tidal volume to levels observed during exercise^20^. The contribution of increased heart rate (HR) is generally ignored, although HR can increase substantially during hyperpnea^31^ and could contribute substantially to the measured increase in energy expenditure. Further, these studies have typically focused on running and other moderate- or high-intensity exercise. The contribution of cardiopulmonary work to walking cost may be higher, as the total cost of walking is considerably lower^32^.

Measuring walking costs under hypoxic conditions provides a potential experimental design for measuring cardiopulmonary costs during exercise. At high altitude, and under normobaric hypoxia, a reduction of alveolar PO_2_ limits pulmonary O_2_ diffusion capability, which induces a decrease in arterial O_2_ saturation (SpO_2_)^33, 34^. Under these conditions, pulmonary ventilation (V_E_) increases within minutes^35^, followed by increases in HR ^36^. These responses are generally considered part of the compensatory mechanisms against hypoxia with reduced oxygen delivery to the peripheral tissues. These results suggest that if hypoxic-induced increases in energy cost of ventilation and/or circulation are observed even at resting conditions, differential energy costs during walking under hypoxia would also be observed.

We developed and tested an experimental model to evaluate the energy cost of ventilation and circulation during walking using acute hypoxic exposure. We used pulmonary ventilation (${{\rm{V}}}_{{\rm{E}}}$) and heart rate (HR) as indices of respiratory and circulatory work, respectively. Healthy male adults, not acclimated to hypoxic or high-altitude conditions, walked over a range of speeds under three different oxygen concentrations (normoxia, moderate, and severe hypoxia) at level gradient. This design enabled us to vary the cardiopulmonary work done while keeping the energy costs to support and move the body (i.e., the mechanical cost of walking) constant across conditions. Our objective was to determine the costs of ventilation and circulation during walking, and to compare values measured during walking to those measured during standing at rest.

## Results

**Figure 1:**
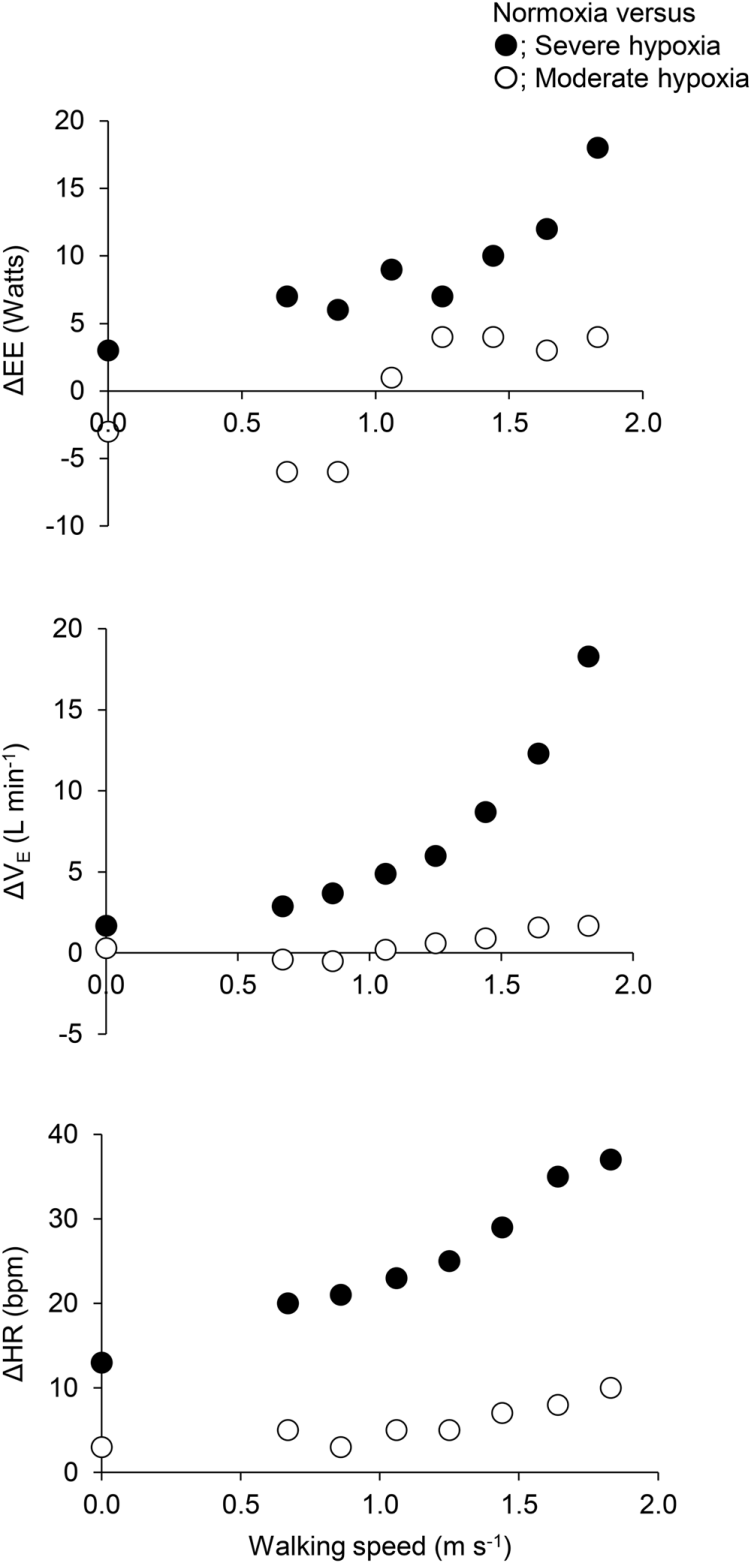
Mean values of differences in energy expenditure (EE: top panel), pulmonary ventilation (V_E_: middle panel), and heart rate (HR: bottom panel) between normoxic trial and moderate hypoxia (open circles), and between normoxic trial and severe hypoxia (filled circles) at rest and each walking speed. The number of 0.0 on the X axis indicate at rest.

Mean (±SD) values for EE, V_E_, and HR at rest and each walking speed, and changes in these variables with hypoxia are given in Table 1. All three change variables, ΔEE, ΔV_E_, and ΔHR, increased with speed and degree of hypoxia, with severe hypoxia having a much stronger effect than moderate hypoxia (Fig. 1). Both walking speed and the degree of hypoxia were significant predictors for ΔEE (model adjusted r^2^ = 0.82, p < 0.001), ΔV_E_ (adj. r^2^ = 0.66, p < 0.001), and ΔHR (adj. r^2^ = 0.92, p < 0.001) in multiple regression.

**Table 1:** Energy expenditure (EE), pulmonary ventilation (V_E_), and heart rate (HR) at rest and each gait speed under three oxygen concentrations. Changes in these variables (Δ) under moderate and severe hypoxia versus normoxia are shown.

	Normoxia	Moderate		Severe	
**EE, Watts**			**ΔEE**		**ΔEE**
Rest	103 ± 18	100 ± 9	−3	106 ± 12	3
0.67 m s^−1^	211 ± 23	205 ± 23	−6	218 ± 20	7
0.86 m s^−1^	230 ± 28	224 ± 23	−6	236 ± 23	6
1.06 m s^−1^	252 ± 34	253 ± 28	1	261 ± 33	9
1.25 m s^−1^	281 ± 34	285 ± 31	4	288 ± 39	7
1.44 m s^−1^	328 ± 50	332 ± 36	4	338 ± 49	10
1.64 m s^−1^	412 ± 64	415 ± 59	3	424 ± 59	12
1.83 m s^−1^	495 ± 83	499 ± 79	4	513 ± 78	18^*^
**V**_**E**_ **,L min** ^**−1**^			**ΔV**_**E**_		**ΔV**_**E**_
Rest	10.9 ± 0.8	11.2 ± 1.4	0.3	12.6 ± 1.4	1.7
0.67 m s^−1^	18.5 ± 2.5	18.1 ± 2.3	−0.4	21.4 ± 3.2	2.9
0.86 m s^−1^	20.2 ± 2.8	19.7 ± 2.7	−0.5	23.9 ± 4.0	3.7^*†^
1.06 m s^−1^	21.8 ± 3.4	22.0 ± 3.4	0.2	26.7 ± 4.9	4.9^*†^
1.25 m s^−1^	24.9 ± 3.1	25.5 ± 4.3	0.6	30.9 ± 6.8	6.0^*†^
1.44 m s^−1^	28.0 ± 3.2	28.9 ± 3.9	0.9	36.7 ± 7.0	8.7^*†^
1.64 m s^−1^	33.3 ± 4.3	34.9 ± 5.9	1.6	45.6 ± 9.4	12.3^*†^
1.83 m s^−1^	40.5 ± 5.3	42.2 ± 8.5	1.7	58.8 ± 15.7	18.3^*†^
**HR, bpm**			**ΔHR**		**ΔHR**
Rest	80 ± 11	83 ± 10	3	93 ± 11	13^*†^
0.67 m s^−1^	78 ± 10	83 ± 8	5	98 ± 11	20^*†^
0.86 m s^−1^	82 ± 10	85 ± 9	3	103 ± 11	21^*†^
1.06 m s^−1^	84 ± 11	89 ± 9	5	107 ± 11	23^*†^
1.25 m s^−1^	89 ± 11	94 ± 9	5	114 ± 10	25^*†^
1.44 m s^−1^	94 ± 10	101 ± 9	7^‡^	123 ± 12	29^*†^
1.64 m s^−1^	101 ± 11	109 ± 8	8^‡^	136 ± 12	35^*†^
1.83 m s^−1^	109 ± 12	119 ± 9	10^‡^	146 ± 14	37^*†^

Values are mean ± standard deviation. bpm; beats per minute. *P < 0.05 between normoxia and severe hypoxia, ^†^ *P* < 0.05 between moderate and severe hypoxia, ^‡^ *P* < 0.05 between normoxia and moderate hypoxia.

**Figure 2:**
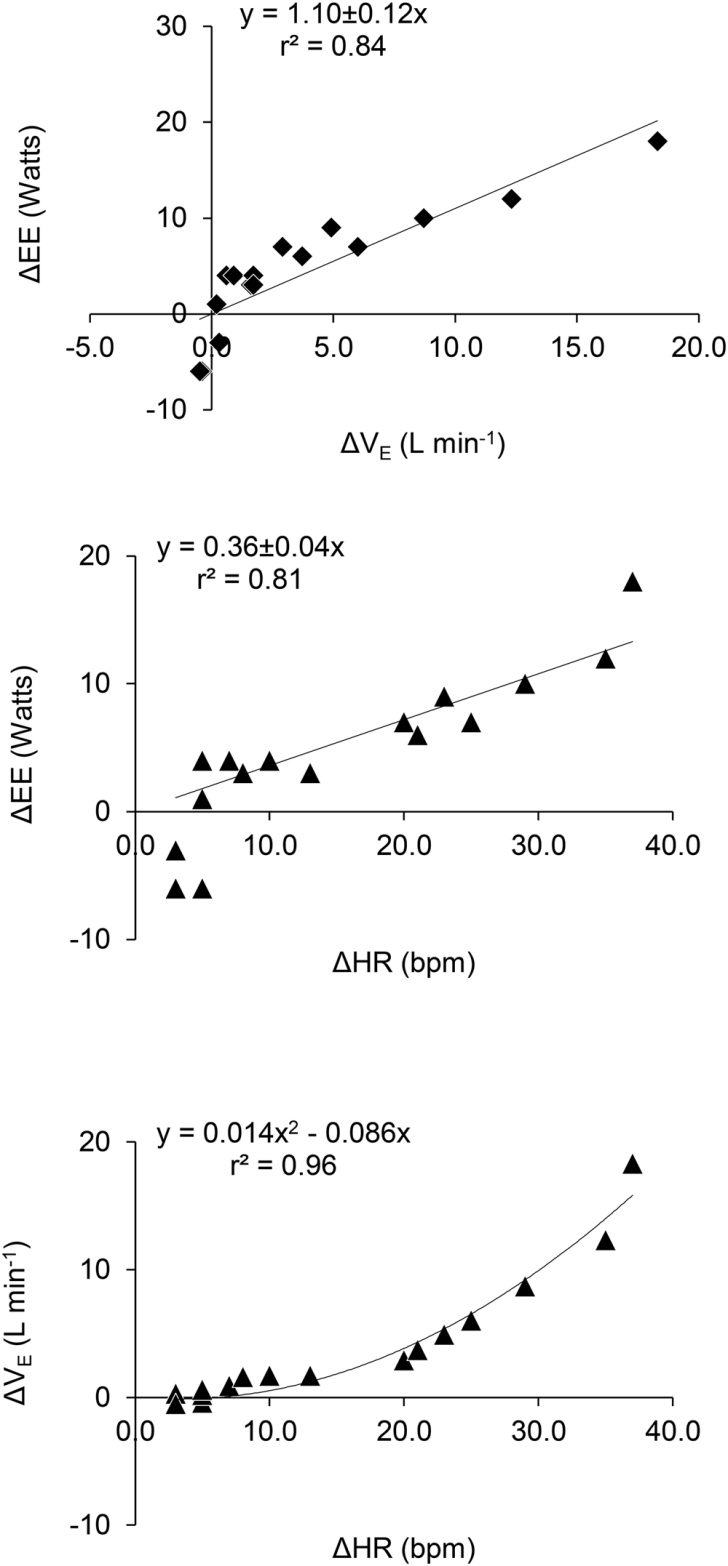
Changes in ΔEE versus in ΔV_E_ (top panel), and changes in ΔEE versus in ΔHR (middle panel). Ordinary least squares linear regression equations, with intercept = 0, are shown. Changes in ΔV_E_ versus in ΔHR (bottom panel). Second order polynomial regression equation, with intercept = 0, are shown.

Bivariate analyses of ΔEE revealed significant correlations with both ΔV_E_ and ΔHR (Fig. 2). With the intercept set at 0, EE increased with ventilatory effort as ΔEE = 1.10 ± 0.12 ΔV_E_ (r^2^ = 0.84, p < 0.001), or roughly 1 Watt for every 1 L min^−1^ increase in V﻿_E_﻿. This cost is equivalent to 3.3 mlO_2_ per L V_E_, assuming 20.1 J per mlO_2_. Energy expenditure increased with circulation as ΔEE = 0.36 ± 0.04ΔHR (r^2^ = 0.81, p < 0.001), or roughly 1 Watt for every 3 beats per minute increase in HR. Slopes changed less than 3% when the three hypoxia-speed conditions with negative ΔEE values were excluded from analysis (Fig. 2).

**Figure 3:**
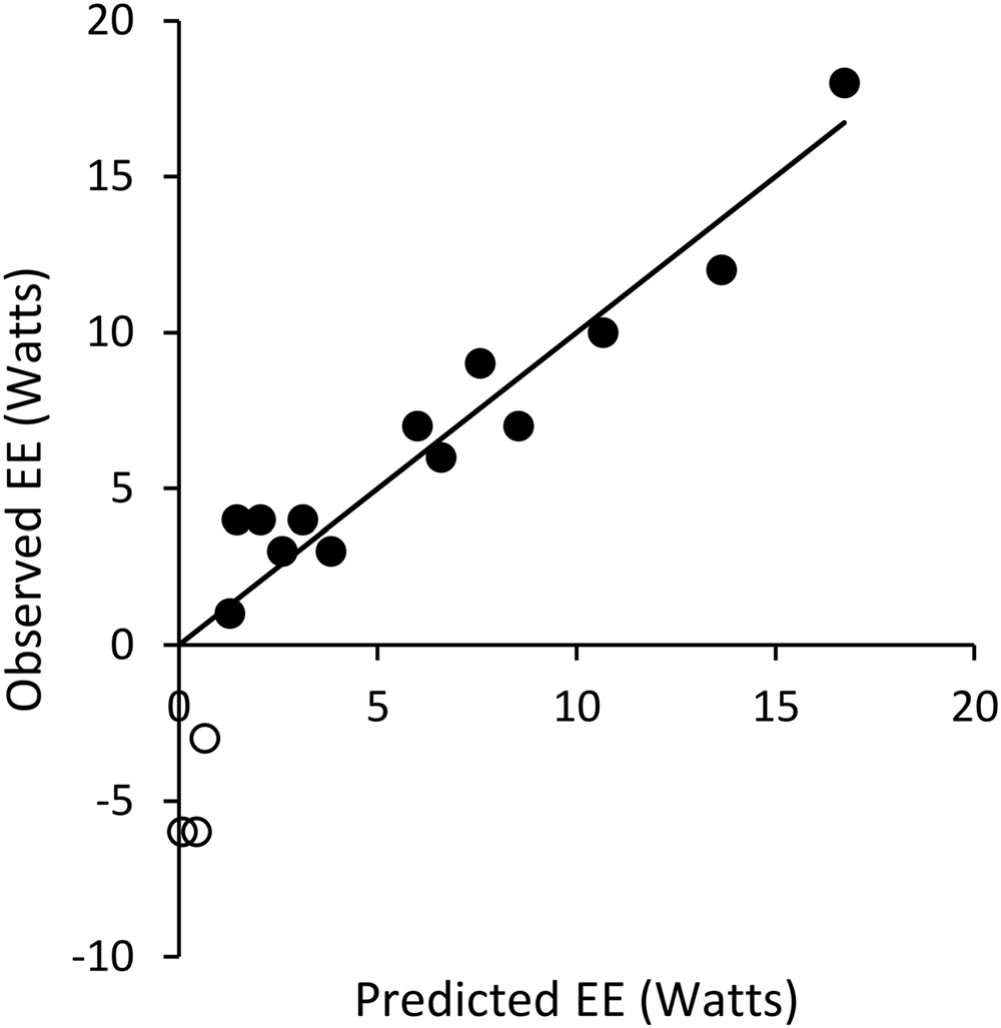
Observed ΔEE plotted against that predicted ΔEE from the least squares regression with V_E_ and HR: ΔEE = 0.44ΔV_E_ + 0.24ΔHR; see text. Line indicates y = x. This predictive fit model for data was calculated using the hypoxia-speed conditions with only positive ΔEE values (filled circles), but excluding the hypoxia-speed conditions with negative ΔEE values (open circles).

ΔV_E_ and ΔHR were strongly correlated (r^2^ = 0.96, second order polynomial regression, Fig. 2), making it difficult to determine their independent effects on EE. When ΔV_E_ and ΔHR were entered as independent variables in a multiple regression, ΔV_E_ achieved significance (t(14) = 2.33, p = 0.036) but ΔHR did not (t(14) = 1.42, p = 0.178). However, when the three hypoxia-speed conditions with negative ΔEE values were excluded from the analysis, both ΔV_E_ (t(11) = 2.87, p = 0.015) and ΔHR (t(11) = 4.65, p < 0.001) were significant, and the fit of the model was very strong (ΔEE = 0.44 ± 0.15ΔV_E_ + 0.24 ± 0.05ΔHR, adj. r^2^ = 0.97, p < 0.001). Notably, this multivariate model reduces the estimated individual costs of ventilation and circulation compared to bivariate analyses. Ventilatory costs are approximately 1 Watt for every 2.3 L min^−1^ increase in V_E_; circulatory costs are approximately 1 Watt for every 4 beats per minute increase in HR. The fit of this predictive model for all data is shown in Fig. 3.

We used the combined model with ΔV_E_ and ΔHR (i.e., EE = 0.44V_E_ + 0.24HR) along with V_E_ and HR values at each condition to estimate the energy cost of cardiopulmonary work at rest and during walking under normoxia, moderate hypoxia, and severe hypoxia. At rest, the estimated energy cost of breathing and circulation accounted for approximately one-fourth of total EE across conditions, rising slightly under severe hypoxia compared to normoxia and moderate hypoxia. As walking speed and EE increased, the percentage of EE attributed to cardiopulmonary work decreased under all conditions. This change was similar in moderate hypoxia and severe hypoxia (Table 2).

**Table 2:** Mean values of EE, the percentage EE to total cardiopulmonary work, and the percentage of net cardiopulmonary EE at rest and each walking speed under all conditions.

	Normoxia	Moderate	Severe
***Watts, W***			
0.67 m s^−1^	26.5	27.5	32.5
0.86 m s^−1^	28.2	28.7	34.8
1.06 m s^−1^	29.4	30.6	36.9
1.25 m s^−1^	31.9	33.3	40.4
1.44 m s^−1^	34.4	36.5	45.1
1.64 m s^−1^	38.4	41.0	52.0
1.83 m s^−1^	43.4	46.5	60.2
***%EE, %***			
0.67 m s^−1^	12.6	13.4	14.9
0.86 m s^−1^	12.3	12.8	14.7
1.06 m s^−1^	11.7	12.1	14.2
1.25 m s^−1^	11.4	11.7	14.0
1.44 m s^−1^	10.5	11.0	13.3
1.64 m s^−1^	9.3	9.9	12.3
1.83 m s^−1^	8.8	9.3	11.7
***% walk net, %***			
0.67 m s^−1^	2.6	2.9	4.5
0.86 m s^−1^	3.6	3.4	5.6
1.06 m s^−1^	3.8	4.0	6.1
1.25 m s^−1^	4.6	4.8	7.1
1.44 m s^−1^	4.8	5.2	7.6
1.64 m s^−1^	4.8	5.2	7.7
1.83 m s^−1^	5.0	5.5	8.0

Next, we subtracted EE and estimated cardiopulmonary expenditure during resting from values measured during walking in order to calculate net walking EE and net walking cardiopulmonary work. Net cardiopulmonary energy expenditure accounted for 3% of net walking EE at 0.67 m s^−1^, rising to 5% at 1.83 m s^−1^ under both normoxia and moderate hypoxia. Under severe hypoxia, these values were slightly higher compared to normoixa and moderate hypoxia. (Table 2).

**Figure 4:**
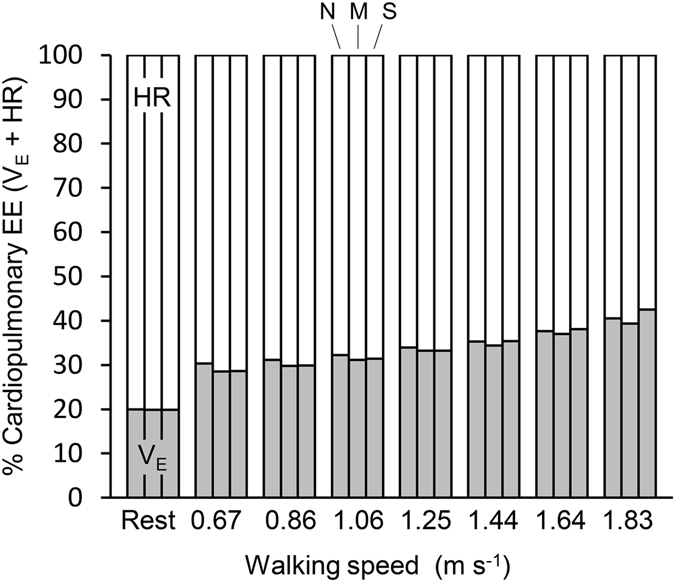
Contribution of ${{\rm{V}}}_{{\rm{E}}}$ (gray segments) and HR (white segments) to cardiopulmonary energy expenditure at rest and at each walking speed under normoxia (N), moderate hypoxia (M), and severe hypoxia (S) conditions.

Finally, we applied the multivariate model with ΔV_E_ and ΔHR to the V_E_ and HR values at each condition to calculate the relative contributions of ventilatory and circulatory work to cardiopulmonary energy expenditure. Across normoxic and both hypoxic conditions, the estimated energy cost of circulation exceeded that of ventilation. Heart beat accounted for 80% of cardiopulmonary work at rest in all three conditions. The proportion of cardiopulmonary work attributable to HR decreased regularly with walking speed under all conditions (Fig. 4).

## Discussion

Both breathing and circulation have measureable effects on EE during both resting and walking. While our findings are consistent with studies using hyperpnea at rest to mimic the costs of ventilation during exercise^20, 22, 23, 25, 29, 30^, results here also indicate that previous work has greatly underestimated the contribution of the heart to cardiopulmonary costs. Our bivariate analysis of V_E_ and EE (Fig. 2) gives a ventilation cost of 3.3 mlO_2_ per L V_E_ (assuming 20.1 J per mlO_2_), consistent with hyperpnea studies reporting 2–4 mlO_2_ per L V_E_ during exercise^20, 26, 30^. However, HR and V_E_ increase together during exercise (Fig. 2) and during enforced hyperventilation at rest^31^. Consequently, analyses that ignore HR and attribute cardiopulmonary costs entirely to breathing will artificially inflate the estimated cost of ventilation. This inflation may be substantial. Ventilation costs decreased 60%, to 1.3 mlO_2_ per L V_E_, and the heart accounted for more than half (60–80%) of cardiopulmonary energy cost, when ventilatory and circulatory costs were considered together in our multivariate model. Future studies using forced hyperpnea to estimate ventilation costs should include simultaneous measures of HR in order to assess the potential contribution of circulatory costs.

Measuring cardiopulmonary costs by induced hypoxia, as done here, enabled us to measure the energy expenditure of the heart non-invasively. To our knowledge, the only previous *in vivo* measures of the heart’s energy expenditure were conducted by monitoring oxygen concentrations in arterial blood supply and venous return of a catheterized heart^37^. Catheterization methods produce estimates of 7 W for heart energy expenditure in adult males lying supine, at rest^37^. Assuming a resting HR of ~60 bpm, this estimate translates to ~7 J per beat. HR cost in our multivariate model was 0.24 ± 0.05 W per bpm, which translates to 14 ± 3 J per beat, twice that of catheterization methods. Given the very different experimental designs, these estimates are reasonably similar. One reason that our approach may produce higher estimates is that our measurements were taken primarily during exercise, which increases blood pressure and stroke volume compared to resting, and presumably increases the energy cost per heartbeat. More work is needed to refine non-invasive estimates of HR cost and to reconcile them with catheterization studies.

The present study focused on walking, rather than the running or cycling tests typical of previous work on cardiopulmonary cost, e.g., refs 20, 22–24, 26, 30. As a result, we examined a relatively low range of V_E_. Maximum V_E_ in the present study was 59 L min^−1^, which falls at the very low end of the range examined in most previous works. The lower range of V_E_ in this study may explain why we did not detect the exponential increase in V_E_ cost at higher ventilation rates reported by those studies^26, 30^.

Cardiopulmonary effort accounts for a substantial portion of resting EE, approximately 23% in normoxic conditions. In contrast, cardiopulmonary energy expenditure accounts for only 3 to 5% of net walking EE (i.e., with resting EE subtracted). The small contribution of cardiopulmonary effort to net walking cost is in line with previous work in humans and a range of other vertebrates^25^. These results lend support to analyses of locomotor cost that focus on musculoskeletal energy expenditure, ignoring cardiopulmonary work, e.g., refs 3, 6, 7, 38–40. Even under severe hypoxia, cardiopulmonary energy cost never exceeded 8% of net walking EE, suggesting that the primary cost of walking at altitude remains the musculoskeletal effort needed to support and propel the body.

Of note, we found small (~3%) reductions in EE with moderate hypoxia compared to normoxia at rest and during walking at slow speeds (0.67 and 0.83 m s^−1^). In our original study^41^, we found that VO_2_ and VCO_2_ in these conditions trended lower (without achieving statistical significance; see Table 1 and Fig. 1 in Horiuchi *et al*.^41^). Similarly, the small reductions reported here for EE, which is calculated from VO_2_ and VCO_2_, do not reach statistical significance. These results are nonetheless unexpected and we can only speculate regarding the cause of this phenomenon, but there are three general possibilities. One is that these small differences are spurious, reflecting measurement error and the limits of precision in our experimental set up. A second possibility is that the body fueled some small percentage of physiological activity using anaerobic metabolism in these conditions. In this case, the aerobic measures of EE employed here would fail to capture some small portion of total energy expenditure in these (and possibly other) conditions, leading to an underestimation of EE and ΔEE. Third, the body may be adjusting to hypoxic conditions by reducing EE to some unspecified set of physiological activities. Given the small size of the effect (~3% or less of EE), we do not expect that this phenomenon greatly affects the results of the currently analyses. Nonetheless, additional research into this phenomenon is warranted.

Another analytical concern here is the effect of CO_2_ stores at the onset of exercise on our measures of VCO_2_, and hence EE. As the generation of CO_2_ increases at the beginning of exercise, initial production will be absorbed in the bloodstream and accumulate there, prior to being exchanged in the lungs and expired. Consequently, measures of expired air, such as those employed here, may underestimate VCO_2_ at the beginning of an exercise bout. However, previous work has demonstrated that the CO_2_ stores were in equilibrium by ~2 min of exercise^42^. As we discarded the first 3 minutes of each trial and used only the last 1 min data of VO_2_ and VCO_2_ at each gait speed in our analyses, the effect of CO_2_ accumulation in the blood should be negligible for this study.

Limitations of this study include the absence of women in our sample and the relatively small range of V_E_, HR, and EE examined. Our multivariate analyses also underscore the inherent difficulties and limitations in parsing the effects of HR and V_E_ on EE, given the physiological linkage between HR and V_E_. Additional work, examining a broader range of subjects and activities, is needed to improve our estimates of HR and V_E_ cost and to determine whether these costs vary with body size, sex, age, or other factors.

Imposing greater cardiopulmonary effort through induced hypoxia provides a non-invasive approach for measuring cardiac and ventilatory costs during locomotion. The advantage of this approach is the ability to measure V_E_ and HR during a given activity, non-invasively, rather than mimicking these costs separately during forced hyperpnea. Results here suggest that previous studies may have overestimated the cost of respiration during locomotion by ignoring the cost of increased HR. Induced hypoxia experiments may prove useful in determining respiratory and circulatory costs across a broader range of activities and conditions.

## Methods

### Participants

The present report represents additional data from investigations that were published previously^41^, examining the interaction of speed and hypoxia on walking cost. Of the 12 subjects who completed the entire protocol in the previous study, 11 completed the experiment in this study. We used the data from these 11 subjects for this study. Subjects (n = 11) were male athletes (sprinters, middle-distance runners, and soccer and baseball players) who engaged in strenuous daily training (2 hours per day, 5–6 days per week). Their mean age, height, and body weight were 24 ± 8 years, 174 ± 6 cm, and 70 ± 9 kg, respectively (values are mean ± standard deviation [SD]). Before this study, we explained all procedures, possible risks, and benefits of participation to the subjects, and all subjects signed an informed consent form. They were asked to refrain from intense physical activity 2 days before testing and to refrain from drinking any alcohol or caffeinated beverages the day before testing. This study was approved by an ethical committee at the Mount Fuji Research Institute according to the Declaration of Helsinki (No: ECMFRI-03-2014).

### Exercise Protocols

All experiments were carried out on a motor-driven treadmill, 2.21 m long and 0.88 m wide (T7000, Johnson Health Tech. Co., Ltd., Taichung Hsein, Taiwan). During an experiment, the subject was allowed to choose his stride and step frequency freely during walking. Subjects wore underwear, shirts, socks, shorts, and lightweight training shoes^43^. They practiced at least three times at several gait speeds to familiarize themselves with treadmill walking while wearing a gas-collection mask. Inspired oxygen concentrations were set at normobaric normoxia (21% room air), moderate hypoxia (FiO_2_; 15%), and severe hypoxia (FiO_2_; 11%). Each trial was performed on a different day in random order with a single-blind study design. First, each subject sat on a chair for 10 minutes, then stood for 5 minutes on the treadmill for baseline measurement. Thereafter, he began to walk on the treadmill. As described in our previous study, seven speeds were set incrementally at 0.67, 0.86, 1.06, 1.25, 1.44, 1.64, and 1.83 m s^−1^, and each speed lasted for 4 minutes^41, 44^.

### Measurements

Pulmonary ventilation (${{\rm{V}}}_{{\rm{E}}}$) and gas-exchange variables were measured with a computerized breath-by-breath system (AE-310S, Minato Ltd., Osaka, Japan). The standard, known gases (O_2_ 15.23%, CO_2_ 4.999%, and N_2_ balance) and room air were used to calibrate the gas analyzer. Inspired and expired gas volumes were measured with a hot wire respiratory flow system. Flow signals were electrically integrated for the duration of each breath to calculate minute ventilation. The expired fractions of O_2_ and CO_2_ were analyzed with a zirconium solid electrolyte oxygen analyzer and an infrared carbon dioxide analyzer, respectively. Each oxygen concentration gas was supplied via a 200-L Douglas bag with a hypoxic gas generator system (Everest summit II, Will Co. Ltd., Tokyo, Japan). HR was measured throughout the study with a commercial HR monitor (POLAR RC800X, POLAR Electro, Tokyo, Japan).

SpO2 was monitored with a pulse oximeter on the left middle finger every 1 minute throughout the study (TM-2564G, A&D, Tokyo, Japan).

### Data Analysis

To calculate energy expenditure, EE, at rest and at each gait speed, ${{\rm{V}}{\rm{O}}}_{2}$ and ${{\rm{V}}\text{CO}}_{2}$ were measured with the following equation.

Energy Expenditure (Watts) = [(3.869 × ${{\rm{V}}{\rm{O}}}_{2}$/1000) + (1.195 × ${{\rm{V}}\text{CO}}_{2}$/1000)] × 4.186/60 × 1000^45, 46^.

At baseline, all physiological values (i.e., gas exchange variables, HR, and SpO_2_) were averaged for the last 2 minutes of standing prior to the start of walking. During walking, a single sample with an average final 1 minute of physiological data was also obtained.

### Calculating Ventilatory and Heart Rate Costs

To evaluate respiratory and circulatory cost, total walking energy expenditure at each speed (including rest) under normoxia (EE_norm_) was subtracted from values under moderate or severe hypoxia (EE_hyp_) to calculate a ΔEE value. We then compared ΔEE to the change in ventilation rate, ΔV_E_ = (V_E hyp_ − V_E norm_) or change in heart rate, ΔHR = (HR_hyp_ − HR_norm_) between conditions. First, we examined the change in EE with respect to V_E_ and HR separately, in a bivariate regression model (Ventilation Cost: ΔEE ~ ΔV_E_. Circulation Cost: ΔEE ~ ΔHR). Next we used multiple regression to test for the independent, additive effects of V_E_ and HR on EE (Cardiopulmonary Cost: ΔEE ~ ΔV_E_ + ΔHR). These regressions were evaluated with the intercept set at 0. This approach assumes that any change in EE between conditions at a given speed is attributable to the change in cardiopulmonary work. This design yielded *n* = 16 comparisons (1 rest + 7 walking speeds for 2 hypoxic conditions).

### Statistics

All data are presented as mean ± SD. A two-way repeated ANOVA (oxygen level × walking speed) was conducted for comparison in EE, V_E_, and HR. As stated above, a bivariate regression model was used for ventilation or circulation cost. Further, multiple regression analysis was also used to test for the independent, additive effects of V_E_ and HR on EE. All statistical analysis were performed using R ver. 2.13.1 (R Core Team, 2014). A *P* value less than 0.05 was considered statistically significant.
